# Neuroprotective Potency of Saffron Against Neuropsychiatric Diseases, Neurodegenerative Diseases, and Other Brain Disorders: From Bench to Bedside

**DOI:** 10.3389/fphar.2020.579052

**Published:** 2020-10-06

**Authors:** Yaqi Bian, Chen Zhao, Simon Ming-Yuen Lee

**Affiliations:** State Key Laboratory of Quality Research in Chinese Medicine and Institute of Chinese Medical Sciences, University of Macau, Macau, China

**Keywords:** saffron, saffron constituents, anti-depressant effect, anxiolytic effect, neuroprotective effects, traditional Chinese medicine

## Abstract

The increasing morbidity rates of brain disorders and conditions such as anxiety, depression, Alzheimer’s disease, and Parkinson’s disease have become a severe problem in recent years. Although researchers have spent considerable time studying these diseases and reported many positive outcomes, there still are limited drugs available for their treatment. As a common traditional Chinese medicine (TCM), saffron was employed to treat depression and some other inflammatory diseases in ancient China due to its antioxidant, anti-inflammatory, and antidepressant properties. In modern times, saffron and its constituents have been utilized, alone and in TCM formulas, to treat neuropsychiatric and neurodegenerative diseases. In this review, we mainly focus on recent clinical and preclinical trials of brain disorders in which saffron was applied, and summarize the neuroprotective properties of saffron and its constituents from chemical, pharmacokinetic, and pharmacological perspectives. We discuss the properties of saffron and its constituents, as well as their applications for treating brain disorders; we hope that this review will serve as a comprehensive reference for studies aimed at developing therapeutic drugs based on saffron.

## Introduction

Brain disorders, i.e. neuropsychiatric and neurodegenerative diseases, have emerged as a majorproblem in recent years. Anxiety and depression are neuropsychiatric disorders that mainly result from intense interpersonal relationships, certain medications, and major stressful life events (divorce or death of a loved one, etc.). Patients suffering from mental disorders usually have symptoms like a decrease or increase in appetite, hypersomnia or insomnia, psychomotor agitation or retardation, and chronic fatigue (Breen et al., 2011). Genetic factors also contribute to the development of depression and anxiety. Chromosome 3p25-26 has been found in more than 800 families with recurrent depression (Pitsikas, 2015). In addition, one of the more common comorbidities is that of anxiety and depression (Bui and Fava, 2017). Accumulating evidence suggest that the underlying pathogenesis of anxiety and depression involve numerous common mechanisms such as control of hormones secretion, functional disturbance of GABAergic system (γ-aminobutyric acid, GABA) (Kalueff and Nutt, 2007) and dysfunction of glutamate-related nervous system (Howells and Russell, 2008; Jia et al., 2020). Furthermore, several signaling pathways involved in the regulations of oxidative stress, neuroinflammation, neurotransmitter dysfunction, and neurotrophic factors (e.g. brain derived neurotrophic factor, BDNF) also contribute to the pathogenesis of anxiety and depression (Kalueff et al., 2006; Ehsanifar et al., 2019). Therefore, treatments targeting these common mechanisms may achieve a more effective therapeutic effect. However, symptoms of depression and anxiety patients sometimes are not exactly the same. For instances, the patients with a diagnosis of major depression are more likely to show a depressed or a sad mood while the patients with a diagnosis of major anxiety mainly display an anxious or a panic mood (Clark et al., 1994). In these cases, the selection of suitable therapeutic strategy becomes more challenging and difficult (Gallagher-Michaels, 2013).

Neurodegenerative diseases are the most prevalent senile diseases in aging populations (especially those aged over 70 years), and include Parkinson’s disease (PD) and Alzheimer’s disease (AD). Cognitive decline, slow and involuntary movements, progressive dementia, and changes of personality are the common symptoms of these two diseases; however, the psychological disorder associated with PD and AD should not be overlooked. Anxiety and depression are secondary changes seen not only in neurodegenerative diseases, but also in other brain disorders. This indicates that the overlap among brain disorders is complex. Since AD and PD are multifactorial disorders without effective cures, nearly all of the drugs on the market aim mainly to alleviate the symptoms (Finley and Gao, 2017). Natural products contain multiple chemical constituents, which are more effective than single chemicals in addressing the pathogenesis of multifactorial disorders through their effects on multiple targets. This explains why drugs developed from natural products with preventive activities against brain disorders are particularly desirable. For example, sodium oligomannate (GV-971^®^) is a marine algae-derived oral oligosaccharide conditionally approved in China for the treatment of mild-to-moderate AD (to improve cognitive function) in November 2019. Unlike most previous anti-PD and anti-AD drugs on the markets, by acting directly on specific target in neuronal cells, GV-971 constitutes a novel agent that therapeutically remodels gut microbiota and suppresses gut bacterial amino acids-shaped neuroinflammation to inhibit AD progression (Wang et al., 2019).

Saffron—the dry red stigma of *Crocus sativus L*—is one of the most expensive herbs on the market today. The flower of *Crocus sativus L* has been widely used as a natural additive in cooking to enhance flavor, color, and aroma. The origin of *Crocus sativus* can be traced back to the Late Bronze Age in Crete; since then, saffron has been cultivated all over the world, but especially in Mediterranean Europe, India, and south-western Asia. Cultivation of saffron requires fertile clay soil and direct sunlight under natural environmental conditions, or greenhouse conditions (which can improve yield) (Galigani and Garbati, 1999; Gresta et al., 2008; Cavusoglu et al., 2009). Saffron is referred to as “red gold,” due to the high market price attributable to hand-harvesting and low production volumes. According to an analysis of food ingredient fraud based on 677 references, saffron is one of the most commonly adulterated products (Moore et al., 2012). Thus, quality control of saffron is important for authentication. For this purpose, various chromatographic and spectrometric methods, such as UV, HPLC, GC, NIR combined with MS, and PTR-TOF-MS have been established and optimized to analyze the components of saffron (Tarantilis et al., 1995; Masi et al., 2016; Grinan-Ferre et al., 2018).

**Figure 1:**
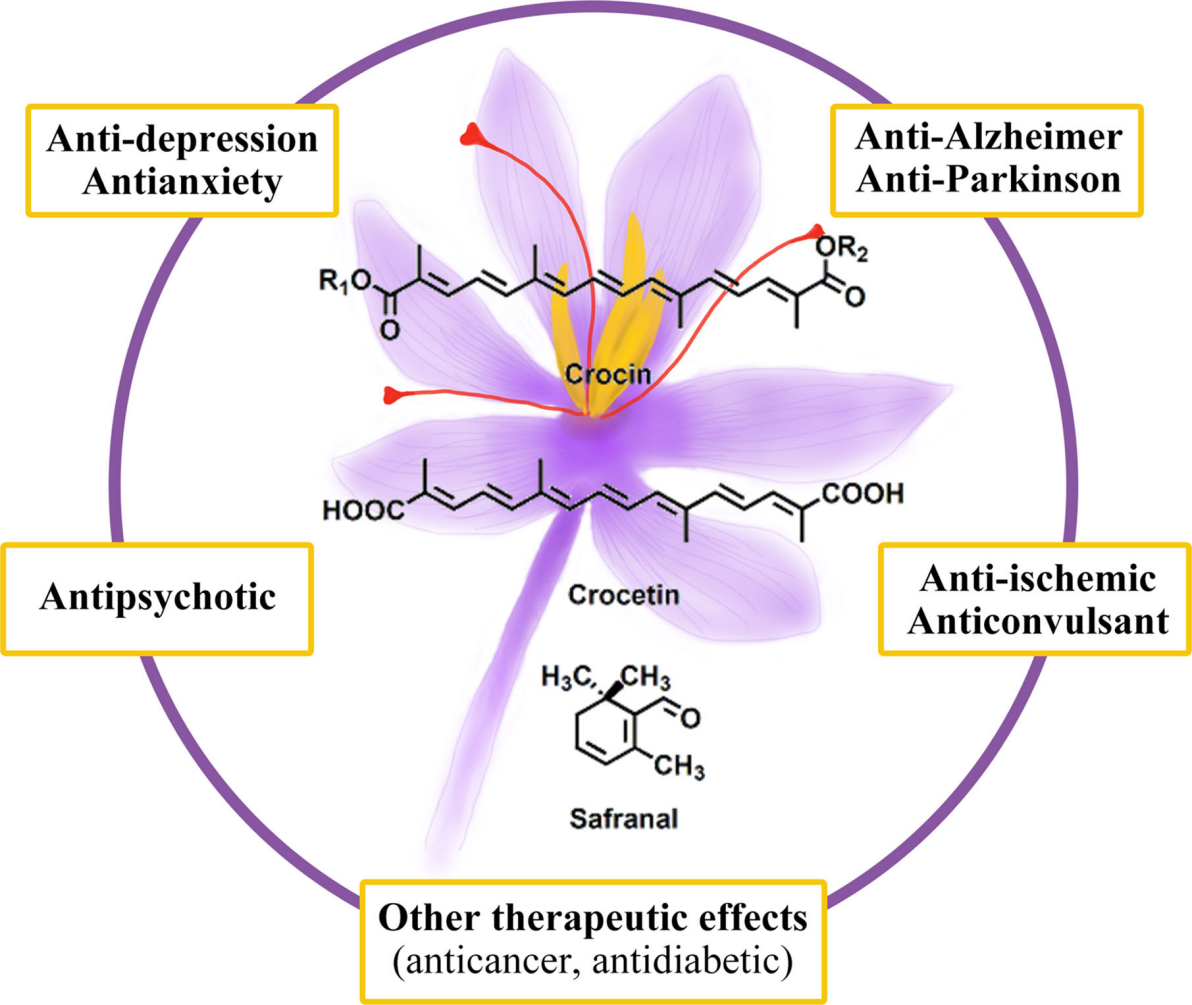
The therapeutic effects of saffron.

Several active ingredients are present in saffron, including carotenoids (crocin, crocetin), monoterpene aldehydes (picrocrocin, safranal), monoterpenoids (crocusatines), isophorones, and flavonoids (Rameshrad et al., 2018). The contents of these active compounds varies from region to region. Saffron has been used in traditional medicine for its hypolipidemic, anti-cancer, antioxidant, anti-inflammatory, and antidepressant properties (Rios et al., 1996). As saffron has pharmacological effects on nervous system, it has also been tested in clinical trials of depression, anxiety, AD, and other brain disorders (Moshiri et al., 2015; Hosseini et al., 2018; Samarghandian and Farkhondeh, 2020). In this review, we summarized preclinical and clinical studies of the use of saffron and its constituents for treating neuropsychiatric diseases, neurodegenerative diseases, and other brain disorders (**Figure 1**).

## Constituents of Saffron

**Figure 2:**
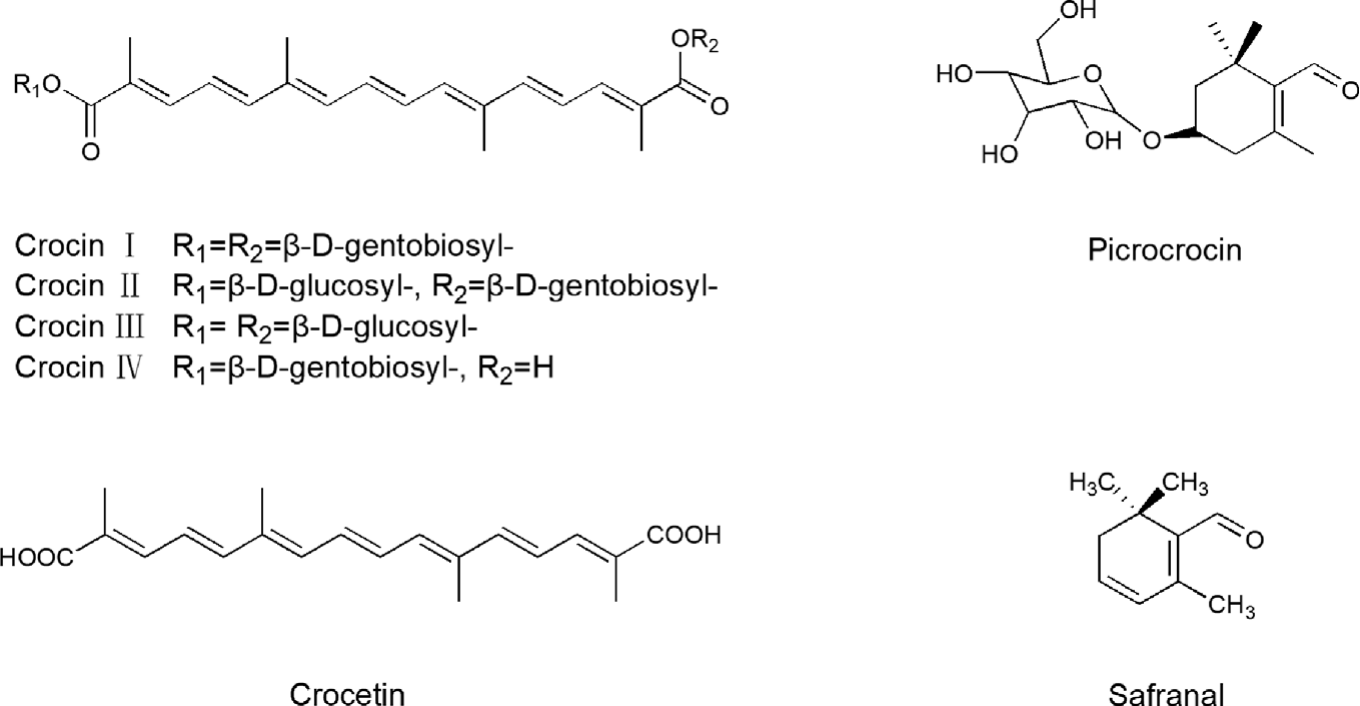
The structural formula of saffron.

Saffron is composed of water, nitrogenous matter, sugars, soluble extract, volatile oil, and fibers, in varying amounts. Among all the components, soluble extract accounts for the highest proportion (41–44%), followed by water (14–16%), sugar (12–15%), and nitrogenous matter (11–13%) (Christodoulou et al., 2015). Saffron contains two vitamins essential to the human body: riboflavin (vitamin B2) and thiamine (vitamin B1). The riboflavin content of saffron ranges from 56 to 138 μg/g, which is the highest amount among all foods (Bhat and Broker, 1953). Apart from these two essential vitamins, small quantities of β‐carotene, essential fatty acids, linoleic and linolenic are also found in saffron. Sterols including campesterol, stigmasterol, and β‐sitosterol have been identified, as well as oleanolic, ursolic, palmitoleic, palmitic, and oleic acids. Most of the volatile compounds are terpenes, terpene alcohols, and their esters. Non-volatile compounds include picrocrocin safranal, crocetin, crocins, and flavonoids (quercetin and kaempferol), among which safranal is the major component (Pitsikas, 2015). About 150 volatile and non-volatile compounds, and nearly 50 constituents, have been identified in saffron (Boskabady and Farkhondeh, 2016). Particularly, the water-soluble carotenoid, crocin, determines saffron’s color. Picrocrocin, the glycoside of safranal, is responsible for its bitterness, while safranal provides the characteristic aroma of saffron. Saffron contains four main bioactive compounds: crocin, crocetin, picrocrocin, and safranal. These four compounds contribute to saffron’s high value and versatility in food and pharmaceuticals. We describe the chemical constituents, neuropharmacological activities, and safety profile of saffron in the following sections (**Figure 2**).

### Crocin

Crocin (8,8’-diapo-8,8’-carotenedioicdioic acid with different glycosides), with a molecular weight of 976.96, is a hydrophilic carotenoid responsible for saffron’s red color. A variety of crocin analogues can be produced *via* the substitution of different glycosyl esters, such as glucose, gentiobiose, and triglucose, into the R1 and/or R2 positions of the side chain (**Figure 2**). As the most abundant crocins in saffron, crocin 1 (or α-crocin) is formed by disaccharide gentiobiose and the dicarboxylic acid crocetin (Samarghandian and Borji, 2014). Qualitative and quantitative analysis of different glycosyl moieties and cis-/trans- isomeric forms of crocins can be aid in the authentication, quality control, standardization, and process traceability of saffron products (Rocchi et al., 2018).

### Picrocrocin

**Figure 3:**
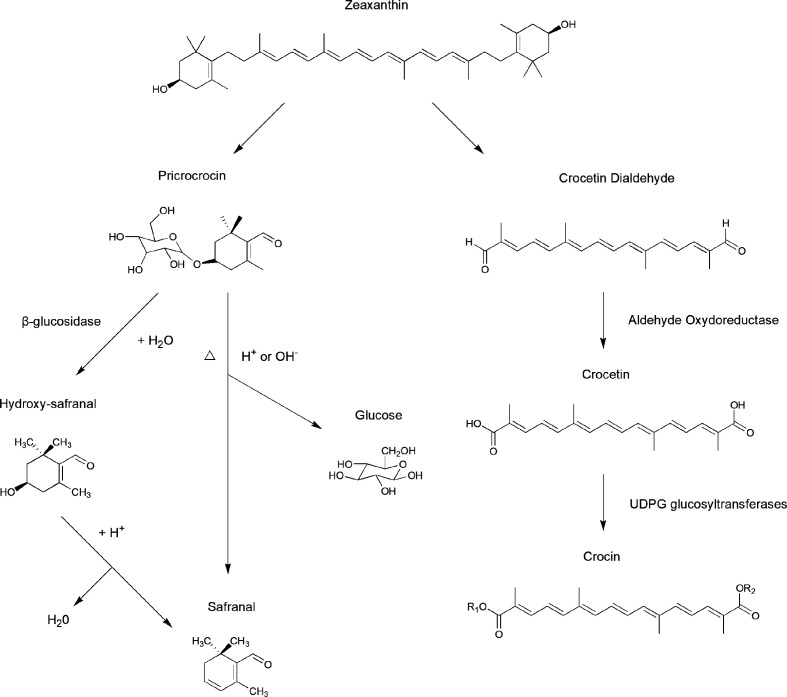
Biosynthesis pathways of crocin, crocetin, safranal, and other important compounds in saffron stigmas.

Picrocrocin (C_16_H_26_O_7_), a crystalline terpene-glucoside of saffron with a molecular weight of 330.37, is the de-glycosylated precursor of saffron’s aromatic components and contributes to its bitter taste (Lage and Cantrell, 2009). Picrocrocin releases hydroxy-safranal (aglycone4−hydroxy−2, 6, 6−trimethyl−1−cyclohexene−1−carboxaldehyde) through the action of β-glucosidase, by dehydration *via* heating and enzymatic reactions occurring in storage. Under natural conditions, safranal is yielded by dehydration during drying (**Figure 3**) (Samarghandian and Borji, 2014).

### Safranal

As the main component of essential volatile oil, safranal (2,6,6-trimethyl-1,3-cyclohexadiene-1-carboxaldehyde) is responsible for the characteristic aroma of saffron. During the dehydration that occurs in post-harvest processing, picrocrocin can produce safranal after being de-glycosylated; thus, the concentration of safranal in saffron is determined by storage time and conditions. As an essential volatile oil, a high safranal content can only maintained for one year after harvesting (Maggi et al., 2010).

## Pharmacokinetics and Safety Evaluation

Crocin and crocetin exhibit very different pharmacokinetic profiles. Crocin can be hydrolyzed to crocetin before (in the gastrointestinal lumen) or during (in the intestinal mucosa) intestinal absorption (Asai et al., 2005). Although crocetin acts as the bioactive compound in rat plasma, oral administration of crocin is preferable to that of crocetin, due to the poor dissolution of the latter substance in intestinal fluid (Zhang et al., 2017). After hydrolysis, crocetin is partly metabolized into mono- and diglucuronide conjugates in the intestinal mucosa (during absorption), liver (after absorption), or both (Asai et al., 2005; Zhang et al., 2017). A clinical pharmacokinetic trial of healthy adult human volunteers showed that, after a single oral administration, crocetin reached a maximum concentration 4 to 4.8 h after administration, and was eliminated with a corresponding half-life of 6.1 to 7.5 h. The results also showed that crocetin exhibited no serious adverse reactions, even up to 22.5 mg. Due to its small molecules and hydrophilic nature, crocetin shows more rapid absorption in the portal vein than in the lymphatics when transported into the bloodstream (Umigai et al., 2011).

After intravenous injection, crocin is converted into crocetin in the gastrointestinal tract. Crocetin has a widespread distribution in tissue and low plasma concentration because of the weak crocetin-albumin interaction. Also, crocetin has therapeutic effects on neurodegenerative diseases due to its ability to penetrate blood-brain barrier (BBB) (Hosseini et al., 2017). To investigate the underlying permeation mechanisms, Lautenschlager et al. established models based on Caco-2 monolayer cells, porcine brain capillary endothelial cells (BCEC), and blood cerebrospinal fluid barrier (BCSFB). Crocin-1 could not penetrate Caco-2 monolayers even at a high concentration of 1,000 μM, which indicates its poor penetration of the intestinal barrier. In contrast, trans-crocetin not only penetrates the intestinal barrier in a dose-dependent manner, but can also gradually permeate the BBB. Instead of the paracellular rout, trans-crocetin is mostly absorbed *via* passive transcellular diffusion (Lautenschlager et al., 2015).

In both experimental and clinical investigations, saffron shows no significant toxicity in therapeutic doses. In acute, sub-acute, sub-chronic, and chronic toxicity tests, no marked changes have been reported in biochemical parameters, hematological parameters, or body organs, among other factors. However, as the key determinant of acute toxicity, the LD_50_ of safranal is lower than that of saffron and crocin, which indicates greater toxicity (Bostan et al., 2017). In a sub-acute toxicity study, although safranal reduced the levels of total cholesterol, triglyceride, and alkaline phosphatase (ALP), it also increased those of lactic acid dehydrogenase (LDH) and serum urea nitrogen (BUN). Moreover, histological results indicate safranal exhibits toxicity in kidney and lung (Hosseinzadeh et al., 2013). In a short-term, double-blinded placebo-controlled clinical trial, saffron tablets were given to patients orally at a dose of 200 or 400 mg for 7 days, and showed an excellent safety (Modaghegh et al., 2008). Crocin tablets were also relatively safe in healthy volunteers at a dose of 20 mg dose for 1 month (Mohamadpour et al., 2013). In studies comparing the efficacy of saffron and placebo in patients with neuropsychiatric diseases, no serious side effects were observed (Mousavi et al., 2015; Mazidi et al., 2016; Lopresti and Drummond, 2017). As the bioactive compounds of saffron can interact with CYP enzymes, drugs with the same function will likely increase the risk of low pharmacological efficacy when co-administrated with saffron (Dovrtělová et al., 2015).

## Pharmacological Actions and Potential Therapeutic Uses of Saffron

### Pharmacological Effects of Saffron on the Central Nervous System (CNS) and Psychological Disorders

#### Depression and Anxiety

##### Depression

Depression is the most prevalent psychiatric disease worldwide, and is associated with high economic costs and a large social burden. Depression is projected to affect up to 21% of the world’s population by the end of 2020 (Murray and Lopez, 1997). Tricyclic antidepressants (TCAs), selective serotonin reuptake inhibitors (SSRIs), and selective serotonin noradrenaline reuptake inhibitors (SSNRIs) are the most commonly used antidepressants. Antidepressants mainly work by increasing the availability of serotonin and certain other neurotransmitters, thereby reducing depressive symptoms (Nelson et al., 2008). Unfortunately, given the lack of precision in targeting symptoms, it is not surprising that the outcomes of nearly all drugs on the market are less than satisfactory. Hence, combination treatments (using serotonergic, noradrenergic, and serotonergic and noradrenergic [and dopaminergic] drugs) involving drugs with two or more mechanisms of action are used to obtain a synergistic effect, or to improve tolerability. Psychotherapy and electroconvulsive therapy are also utilized as adjuvant therapeutic measures to improve the efficacy of drug treatment (Moret, 2005). Typical side effects include insomnia, somnolence, dry mouth, constipation, and tachycardia. Low rates of full remission, prolonged delays in symptom resolution, substantial residual symptoms after treatment, and high relapse rates are the major problems associated with currently available antidepressants (Si and Yu, 2016). Promisingly, some natural products have antidepressant effects, such as saffron, resveratrol, green tea catechins, cocoa, omega-2, anthocyanins, and B vitamins. Therefore, new drugs developed from extracts of natural products, especially those that have been shown to have low side effects in the treatment of depression, are becoming increasingly desirable (Siddiqui et al., 2018).

###### Preclinical Studies

Extracts of saffron (aqueous and ethanolic) were demonstrated to have antidepressant effects in a rodent depression model. Hosseinzadeh et al. confirmed the antidepressant effect of saffron in a forced swimming test completed by mice. The results showed that safranal (0.15–0.5 mg/kg), crocin (50–600 mg/kg), and the extracts of saffron stigma (0.2–0.8 g/kg) reduced the immobility time compared to the saline group. Swimming time was increased by both the extracts and safranal, in a manner comparable to fluoxetine. This indicated that the underlying mechanism may involve the activation of dopaminergic, noradrenergic, and serotonergic systems (Hosseinzadeh et al., 2004). In a similar study, also conducted by Hosseinzadeh, another constituent of saffron, kaempferol, had positive effects in both mice and rats depression models. In another preclinical study, Wang et al. confirmed therapeutic effects of saffron on depression. In this study, the aqueous ethanol extract of saffron was fractionated, based on the polarity at which the petroleum ether fraction and dichloromethane fraction showed dose-dependent antidepressant effects in a behavioral model of depression (Wang et al., 2010). Amin et al. discovered that crocetin has a stronger antidepressant effect than crocin, since a higher dose of the latter was needed in acute and sub-acute administration regimens (Amin et al., 2015). Several other studies found that aqueous extracts of saffron showed antidepressant effects in various experimental depression models that involved modulation of the BDNF, CREB, and VGF pathways (Dorri et al., 2015; Ghasemi et al., 2015; Razavi et al., 2017; Asrari et al., 2018). Moreover, crocin has exhibited anti-inflammatory effects by suppressing the expression of NF-kB and NLRP3 signaling pathway activity in an LPS-induced mouse model; neuroinflammation has been suggested to be a potential mechanism (Zhang et al., 2018).

###### Clinical Studies

Due to the efficacy of saffron in the treatment of depression demonstrated in many preclinical studies, several clinical trials on saffron have been performed over the last few decades. Moshiri et al. and Akhondzadeh et al. obtained the same result, i.e., that that patients who received saffron 30 mg/day (*b.i.d*.) for 6 weeks showed better outcomes according to the Hamilton Depression Rating Scale than patients who received placebo (*b.i.d*.). Both studies included 40 outpatients who met the DSM-IV (Diagnostic and Statistical Manual of Mental Disorders-IV) criteria for depression and received either saffron or placebo treatment (Akhondzadeh et al., 2005; Moshiri et al., 2006). Another clinical trial showed that saffron can reduce depression severity. That 4-week study involved 40 patients suffering from major depression according to the DSM-IV criteria; the patients were randomly assigned to a fluoxetine and saffron group or fluoxetine and placebo group. Even though depression severity was reduced in both groups, at the end of the study there were no significant group differences, indicating no additional benefit of saffron taken in conjunction with fluoxetine. Further investigation of longer-term treatment outcomes is merited (Sahraian et al., 2016). In another randomized double-blind study, Lopresti and colleagues observed 123 patients for 12 weeks. They concluded that various doses of curcumin and combined curcumin/saffron treatment can reduce depressive and anxiolytic symptoms in patients with major depressive disorder. Similarly, Mazidi et al. found that, compared with the placebo group, patients taking 50 mg saffron (*b.i.d*.) for 12 weeks showed an improvement in anxiolytic and depressant symptoms (Mazidi et al., 2016; Lopresti and Drummond, 2017). Postpartum depression is a subtype of depression that can affect new mothers after childbirth. A randomized, double-blind placebo-controlled trial was conducted on 60 women suffering from postpartum depression. At the final assessment, 96% patients in the saffron group were in remission compared to 43% in placebo group. The complete response rate of the saffron group reached 60%, which was higher than that of the placebo group (Tabeshpour et al., 2017). In addition, several clinical studies have been carried out to evaluate the antidepressant effects of the active ingredient in saffron, crocin. Talaei et al. reported that crocin combined with one SSRI (fluoxetine, sertraline or citalopram) had a greater antidepressant effect than SSRI combined with placebo (Talaei et al., 2015).

As well as clinical studies comparing the antidepressant effects of saffron and placebo, several studies compared saffron with clinical antidepressants. Shahmansouri et al. and Noorbala et al. both compared saffron and fluoxetine, in terms of therapeutic effects on depression, in a 6-week study. In both studies, saffron had a comparable therapeutic effect to that of fluoxetine on mild-to-moderate depression. In a pilot double-blind randomized trial, Akhondzadeh et al. reported a similar result to the two studies mentioned above, with no side effects (Noorbala et al., 2005; Akhondzadeh Basti et al., 2007; Shahmansouri et al., 2014). A double-blind, randomized clinical trial found that, after receiving saffron (15 mg capsule, *b.i.d*.) or fluoxetine (20 mg capsule, *b.i.d*.), nearly 50% of the patients in both groups experienced an improvement of depressant symptoms and a reduction in depression scores. There were no significant differences between the groups (Kashani et al., 2017). Two other studies compared the antidepressant effects of saffron with either imipramine or citalopram. Despite the similar results to those reported above, saffron exerted fewer adverse effects compared with imipramine (Akhondzadeh et al., 2004; Ghajar et al., 2017). Thus, substantial clinical evidence indicates that saffron is an effective alternative solution to antidepressant drugs for the management of depression.

##### Anxiety

Anxiety is a serious psychiatric condition that can manifest as panic disorder, phobias such as agoraphobia or claustrophobia, social anxiety disorder etc.; anxiety affects more than 6% of the world’s population. SSRIs, serotonin, and noradrenaline reuptake inhibitors (SNRIs), and pregabalin are still the first-line drugs recommended by international guidelines. However, side effects, delayed action, and worsening of anxious symptoms at the beginning of treatment make it hard to achieve an ideal therapeutic outcome and tend to preclude continuous treatment. Meanwhile, natural products such as bacopa monniera, centella asiatica, galphimia glauca and matricaria recutita etc. have demonstrated anti-anxiety effects (Sarris, 2018). New drugs synthesized from natural products may have a bright future owning to fewer side effects and a shorter onset time (Maron and Nutt, 2017).

###### Preclinical Studies

Hosseinzadeh et al. compared the anxiolytic and hypnotic effects of saffron extract, crocin, and safranal using an elevated plus maze test in a mouse model of anxiety. The results showed that saffron aqueous extract and safranal, but not crocins, had anxiolytic and hypnotic effects (Hosseinzadeh and Noraei, 2009). Another study obtained similar results in an animal model of anxiety using a light/dark test. However, there were differences from the experiments conducted by Hosseinzadeh et al., in that both crocin and diazepam could increase the “darkness entering latency of rats” in a light/dark test, suggesting that crocin had anxiolytic-like effects (Pitsikas et al., 2008). Ghalandari-Shamami et al. exposed rats to stress during adolescence to evaluate the anxiolytic effects of crocin and physical activity (voluntary wheel running exercise). Crocin, physical activity, and the combined intervention all alleviated the behavioral and morphological deficits induced by adolescent stress (Ghalandari-Shamami et al., 2019). Other studies investigated the therapeutic effects of crocin on obsessive-compulsive disorder and stress-induced anorexia. The results showed that both crocin and aqueous extracts of saffron had the ability to attenuate symptoms, albeit to differing extents (Halataei et al., 2011; Georgiadou et al., 2012).

###### Clinical Studies

Several randomized double-blind clinical trials have been performed to evaluate the efficacy of saffron or saffron extracts on anxiety. Another 6-week study, involving 66 patients who suffered from major depression accompanied with anxiety, compared the anxiolytic effects of saffron (30 mg/day) and citalopram (40 mg/day). Obvious improvement of anxiety symptoms was observed and no severe side effects were seen in either group (Ghajar et al., 2017). Two clinical trials were designed to investigate the anxiolytic effect of affron^®^ (a novel saffron extract) on both adults and youths. In the first study, Kell et al. found that affron^®^ (28 mg/day for 4 weeks) notably improved anxiety-like symptoms in healthy adults (Kell et al., 2017). The second trial was focused on youths (aged 12–16 years) with mild-to-moderate anxiety or depression symptoms. The results showed that administration of affron^®^ (14 mg, *b.i.d*.) for 8 weeks improved anxiety and depressive symptoms in the youths (Lopresti et al., 2018). Furthermore, Milajerdi et al. investigated whether saffron has a therapeutic effect on mild-to-moderate depression-anxiety in type 2 diabetes mellitus (DM) patients. Anxiety and sleep disturbance in the DM patients were relieved after 8 weeks of treatment with saffron (Milajerdi et al., 2018). However, a 12-week, double-blind randomized placebo-controlled clinical trial obtained different results. Men and women with on-pump coronary artery bypass grafting (CABG) were included in the study and received either saffron capsules (15 mg/twice daily) or placebo. The results did not support the hypothesis of a therapeutic effect of saffron in post-CABG patients with symptoms of depression and anxiety. The limitations of the study included a small sample size, short study duration, and non-comprehensive study design (Moazen-Zadeh et al., 2018).

#### Alzheimer’s Disease and Parkinson’s Disease

##### Alzheimer’s Disease

AD is a slowly progressing neurodegenerative disease associated with progressive loss of learning and memory function. Pathological changes, such as the formation of neurofibrillary tangles (NFT) and amyloid plaques, can cause a range of biological dysfunctions in the brain, ultimately leading to memory and learning ability loss. AD is one of the most common causes of dementia, especially in elderly populations. There will be approximately 50 million people with dementia associated with AD by 2040 (Finley and Gao, 2017). Thus, new drugs are urgently needed for the treatment of AD. Natural products are currently the “hot topic” in neurodegenerative diseases. Regarding its therapeutic effects on brain disorders, saffron has been proven to alleviate the symptoms of AD.

Depression and anxiety are two frequent and challenging comorbidities of AD. When accompanied by personality changes, depression and anxiety are often neglected and negatively impact quality of life. Studies have shown that patients with severe AD were more likely to have depression. Moreover, depression and anxiety could accelerate the progression, and increase the mortality, of AD patients. Patients are likely to benefit from saffron because of its antidepressant and anti-anxious effects (Van der Mussele et al., 2013; Chi et al., 2015; Gracia-Garcia et al., 2015).

###### Preclinical Studies

Saffron and its constituents have neuroprotective effects against chemically induced cognitive impairment in experimental animal models (Dashti et al., 2012; Naghibi et al., 2012; Naghizadeh et al., 2013; Asadi et al., 2015; Ghaffari et al., 2015). Moreover, amyloid-β (Aβ) peptide, phosphorylated tau proteins, and their associated signaling pathways are potentially crucial therapeutic targets for AD intervention. The neuroprotective effects of crocin and crocetin were demonstrated by several *in vitro* studies. The results showed that both crocin and crocetin could provide neuroprotection by reducing Aβ aggregation, phosphorylated tau formation, and synaptic loss. AD is an intractable neurodegenerative disease and complex mechanisms regulate its progress. Several studies have proved that saffron and its constituents, especially crocin and crocetin, achieve a neuroprotective effect *via* attenuating oxidant stress, endoplasmic reticulum stress, neuroinflammation, damage of the BBB, and neuronal cell apoptosis (Papandreou et al., 2006; Ahn et al., 2011; Deslauriers et al., 2011; Ghahghaei et al., 2012; Ghahghaei et al., 2013; Kong et al., 2014; Karakani et al., 2015; Rashedinia et al., 2015). A recent study showed that the neuroprotective effects of saffron may involve the MAPK and PI3K pathways (Rafieipour et al., 2019).

###### Clinical Studies

Since there are no effective drugs for AD, natural products are being emphasized in the development of therapeutics. Few clinical studies have compared the effects of saffron with either first-line drugs or placebo. Tsolaki et al. reported that saffron could be a good choice for management of mild cognitive impairment, where it improved Mini-Mental State Examination scores in patients (Tsolaki et al., 2016). To compare the effects of saffron with the first-line drugs used by AD patients, Akhondzadeh et al. conducted a clinical trial. The results showed that saffron (30 mg/day) was as effective as donepezil (10 mg/day) for mild-to-moderate AD patients. Another double‐blind, randomized study compared saffron with memantine in terms of their ability to alleviate cognitive impartment. In that study, saffron was comparable to memantine in terms of reducing cognitive decline in AD patients. It has also been reported that saffron exerts synergistic effects with other nutraceuticals (Bacopa monnieri, l-theanine, copper, folate, and vitamins of B) to influence cognitive function (Akhondzadeh et al., 2010a; Akhondzadeh et al., 2010b; Farokhnia et al., 2014; Cicero et al., 2017).

##### Parkinson’s Disease

PD is a common neurodegenerative disease characterized by the loss of dopaminergic neurons in the substantia nigra pars compacta. The symptoms of PD include tremor, bradykinesia, rigid muscles, impaired balance, and loss of automatic movements. Preclinical results indicate that saffron may be a promising target for curative drugs for PD (Pan et al., 2016).

PD in the early stages may manifest as non-motor symptoms such as sleep disorder, depression, and anxiety. In some cases, non-motor symptoms may even be the first symptoms. Studies have shown that the morbidity of depression in patients suffering from PD ranges from 2.7 to 90% due to differences in diagnostic criteria and study populations. According to epidemiological data, nearly 97% of PD patients have two or more non-motor symptoms, including anxiety. It is clear that the severity of PD is positively correlated with the development of non-motor symptoms. Saffron deserves more attention as a potential therapeutic for PD given its antidepressant and anxiolytic effects (Arabia et al., 2007; Pontone et al., 2009; Schrag and Taddei, 2017; Ryan et al., 2019).

###### Preclinical Studies

Saffron has been shown to exert multiple neuroprotective effects in different disease models. Abdullah et al. found that crocetin, one of the constituents of saffron, exerted neuroprotective effects in a 6-OHDA-induced rat PD model by attenuating oxidative stress (Ahmad et al., 2005). In another study, saffron exerted a neuroprotective effect on nigral and retinal dopaminergic cells in MPTP-treated mice (Purushothuman et al., 2013). Guo-Feng Zhang et al. showed that another constituent of saffron, crocin, protected pheochromocytoma (PC-12) cells against MPP^+^-induced injury through inhibiting mitochondrial dysfunction and ER stress (Zhang et al., 2015). Crocin was found to improve motor deficits and reduce inflammatory cytokines in a malathion-induced rat model (Mohammadzadeh et al., 2018). The antioxidative and antiapoptotic effects of safranal were investigated in an *in vitro* model of rotenone-induced PD. Safranal protected primary dopaminergic cells against oxidative stress and apoptosis *via* the Keap1/Nrf2 signaling pathway (Pan et al., 2016). Saffron and its constituents crocin and crocetin were also shown to exert neuroprotective effects by inhibiting the aggregation and accumulation of α-synuclein (Inoue et al., 2018). In a study conducted by Tamegart et al., saffron reversed dopaminergic and noradrenergic damage induced by lead (Tamegart et al., 2019). As well as in animal and cell models, neuroprotective effects of saffron and crocin were also confirmed by Rao et al. in a drosophila model of parkinsonism (Rao et al., 2016).

### Other Brain Disorders

#### Post-Traumatic Stress Disorder (PTSD)

Post-traumatic stress disorder (PTSD) is a mental disorder, which is caused by experiencing or witnessing a catastrophic incident such as natural disaster, war, serious accident, assault, rape, and abuse. Flashback of trauma, avoidance of certain places, feeling tense, insomnia, and nightmares are the most common symptoms occurred in PTSD patients (Auxemery, 2018). Hormones changes, such as the increase of adrenaline, vasopressin, and corticotropin-releasing hormone (CHR), are generally considered to be responsible for the cause of the psychological disorders including different types of anxiety and depression (Newport and Nemeroff, 2000; Asalgoo et al., 2015). The recommended standard treatments for PTSD patients are psychological therapies (e.g. cognitive behavioral treatment, and talk therapy), medications (e.g. antidepressants, and cannabinoids), or combination of different methods (Watson, 2019).

Some preclinical studies on testing the effects of saffron and its constituents on PTSD animals have obtained encouraging results. Iranian scientists found out that both saffron extract and crocin could significantly reduce the plasma corticosterone level as well as the anorexic time in a PTSD rat model (Sahraei et al., 2012). Asalgoo et al. and his colleague reported that saffron extract and crocin could enhance the ability of spatial learning and attenuate the freezing behavior also in a PTSD rat model (Asalgoo et al., 2018). Regarding management of anxiety behavior, another study showed that combination oral intake of saffron and deep brain stimulation (DBS) exhibited a more efficient therapeutic effect than monotherapy of DBS (Hashtjini et al., 2018). All these promising pre-clinical results shall be further validated in clinical study in PTSD patients.

#### Schizophrenia

Schizophrenia is a severe mental disorder characterized by abnormal behavior, strange speech, a decreased ability to understand reality, and social, occupational, and individual dysfunction. The aetiology and pathophysiology of schizophrenia remain unknown. The complexity of schizophrenia is reflected in the different types of enduring and persistent psychotic symptoms (positive symptoms, negative symptoms, and cognitive disturbances). Because of the complexity of schizophrenia, current antipsychotic drugs have shown efficacy only for positive symptoms, such as hallucinations, delusions, catatonic behavior. There are no effective drugs for the negative symptoms (social withdrawal, anhedonia, avolition) or cognitive disturbances (deficits in attention and memory) (Pitsikas, 2016).

Few studies have investigated the effects of saffron in schizophrenia-like models. Georgia Georgiadou et al. found that crocin (50 mg/kg, *i.p.*) could attenuate the hypermotility, stereotypies, and ataxia induced by ketamine. Moreover, crocin (50 mg/kg, *i.p.*) counteracted ketamine-induced social isolation in the social interaction test (Georgiadou et al., 2014). Using a novel object recognition task (NORT), another study showed that crocin (15 and 30 mg/kg) reversed recognition memory deficits induced by apomorphine in rat schizophrenia-like models (Pitsikas and Tarantilis, 2017). Two clinical studies used saffron in schizophrenia patients but only investigated its effects on non-schizophrenia symptoms. Fadai et al. reported that saffron extract could alleviate metabolic syndrome, while Mousavi et al. found that saffron aqueous extract and crocin (15 mg *b.i.d*.) had no side effects in patients suffering from schizophrenia (Fadai et al., 2014; Mousavi et al., 2015).

#### Epilepsy

Epilepsy is characterized by abnormal hypersynchrony of neuronal activity due to an imbalance between glutamatergic signaling pathway-mediated excitatory neurotransmission and the GABAergic signaling pathway. Epilepsy constitutes a highly significant health concern and financial burden that affects about 50–65 million people worldwide. The main symptom of epilepsy is recurrent seizures (Eyo et al., 2017).

It was shown that safranal could reduce seizure duration and delay the onset of tonic convulsions (Hosseinzadeh and Talebzadeh, 2005; Hosseinzadeh and Sadeghnia, 2007). In addition, a study found that safranal exerted anticonvulsant activity through the GABA_A_-benzodiazepine receptor complex and might have interact with opioid receptors (Hosseinzadeh and Sadeghnia, 2007). Results obtained by Iranian researchers showed that administration of crocin (100 μg) had a comparable anticonvulsant effect to diazepam (10 μg) in a penicillin-induced epilepsy rat model, indicating involvement of the GABAA-benzodiazepine receptor (Tamaddonfard et al., 2012). In another study, crocin (5, 10, and 20 mg/kg *p.o.*) improved cognitive impairment in male Swiss albino mice by suppressing ROS generation and NF-κB pathway signaling (Mazumder et al., 2017). Additionally, hydroethanolic saffron extract (CSE) (10–200 μg/ml) inhibited evoked postsynaptic potentials (PSPs) and decrease glutamate-induced membrane depolarization (Berger et al., 2011). Other researches have shown that aqueous and ethanolic extracts of *Crocus sativus L*. stigma may benefit both absence and tonic clonic seizures (Hosseinzadeh and Khosravan, 2002).

#### Stroke

Stroke is one of the major causes of morbidity and mortality in developed and developing countries. Increasing evidence indicates that oxidative stress, inflammation, mitochondrial dysfunction, and excitotoxicity in ischemic areas account for the pathogenic progression of stroke (Luo et al., 2019). Oxidative stress is particularly implicated in stroke, and is one of the causes of dysfunction and death of neuronal cells (Barnham et al., 2004). Therefore, drugs that target oxidative stress may be useful in the treatment of stroke.

As a potent antioxidant, crocin has the ability to prevent the death of PC-12 cells by suppressing the generation of ROS (Ochiai et al., 2004; Ochiai et al., 2007). By the same token, saffron extract (Saleem et al., 2006), crocin (Ochiai et al., 2007; Zheng et al., 2007; Vakili et al., 2014), crocetin(Higashino et al., 2014), and safranal (Hosseinzadeh and Sadeghnia, 2005; Sadeghnia et al., 2017) exerted protective effects against ischemic injury by ameliorating excessive oxidation and increasing antioxidant activities in rat and mouse models. Neuroprotective effects of crocin were attributed to the regulation of MDA, SOD, GPx, and the c-jun kinase (JNK) pathway in the ischemic cortex (Ochiai et al., 2007; Vakili et al., 2014). In a recent randomized clinical trial, patients with acute ischemia stroke were randomly divided into two groups and subjected to either routine stroke care or routine stroke care with saffron capsule treatment (200 mg/day), for a 3-month follow-up observation. Based on the Institute of Health Stoke Scale (NIHSS), the severity of stroke was significantly alleviated in saffron-treated group during the first 4 days. Decreased serum neuron specific enolase, s100 and increased BDNF were also observed in saffron-treated group. At the end of this trial, patients in saffron-treated group showed a higher mean Barthel index, which measures functional independence and mobility in patients with chronic and disabling conditions, than patients in control group (Asadollahi et al., 2019).

## Conclusion

As one of the most expensive spices in the world, saffron and its constituents, such as crocin, crocetin, and safranal, have shown various biochemical and pharmacological functions. In this comprehensive review, we aimed to summarize the chemical profiles, pharmacological activities, and therapeutic applications of saffron and its constituents in diseases of the central nervous system. Both preclinical and clinical trials suggested that saffron was effective and safe without serious side effects. According to current scientific evidence, saffron and its bioactive compounds have multiple therapeutic effects in many conditions, including psychological disorders, neurodegenerative diseases, cancer, diabetes, and cardiovascular diseases. Preclinical studies proved that saffron exerts its neuroprotective effects mostly *via* antioxidative stress, anti-neuroinflammation, anti-apoptosis and certain other related pathways. Clinical trials also confirmed that saffron could alleviate depressive and anxiety-like symptoms in both depression and anxiety patients. Improvement of cognition impairment was observed in clinical studies using saffron for treating neurodegenerative diseases such as AD and PD. Taken together, the findings provide a fresh perspective that could aid the development of novel neuroprotective drugs from saffron and its bioactive compounds. More investigation of saffron is needed, including preclinical and clinical studies, in terms of its potential to treat neuropsychiatric diseases.

## Author Contributions

YB and CZ contributed equally to this work. All authors contributed to the article and approved the submitted version.

## Funding

This study was funded by The Science and Technology Development Fund, Macau SAR (File no. 0058/2019/A1 and 0016/2019/AKP), and University of Macau (File no. MYRG2019-00105-ICMS).

## Conflict of Interest

The authors declare that the research was conducted in the absence of any commercial or financial relationships that could be construed as a potential conflict of interest.

## Abbreviations

PD, Parkinson’s disease; AD, Alzheimer’s disease; BBB, blood-brain barrier; BCEC, brain capillary endothelial cells; BCSFB, blood cerebrospinal fluid barrier; ALP, alkalin phosphatase; LDH, lactic acid dehydrogenase; BUN, serum urea nitrogen; CNS, central nervous system; TCAs, tricyclic antidepressants; SSRIs, selective serotonin reuptake inhibitors; SSNRIs, selective serotonin noradrenaline reuptake inhibitors; SNRIs, noradrenaline reuptake inhibitors; BDNF, Brain Derived Neurotrophic Factor; CREB, cAMP-response element binding protein; VGF, vascular growth factor; NF-kB, nuclear factor-kappaB; NLRP3, NOD-like receptor family pyrin domain-containing 3; LPS, lipopolysaccharide; DSM-IV, Diagnostic and Statistical Manual of Mental Disorders-IV; CABG, Coronary Artery Bypass Grafting; NTF, neurofibrillary tangles; MAPK, Mitogen-activated protein kinase; PI3K, phosphatidylinositol 3-kinase; 6-OHDA, 6-hydroxydopamine; MPTP, 1-Methyl-4-phenyl-1,2,3,6-tetrahydropyridine; MPP+, 1-methy1-4pheny1-pyridine; Nrf2, NF-E2-related factor 2; Keap1, Kelch-like ECH-associated protein 1; NORT, novel object recognition task; ROS, Reactive oxygen species; H2O2, hydrogen peroxide; NO, nitric oxide; MDA, malondialdehyde; TBARS, thiobarbituric acid reactive substances; SOD, superoxide dismutase; GPx, glutathione peroxidase; GSH, glutathione peroxidase; MDA, malondialdehyde; Glu, glutamate; Asp, aspartate; CAT, catalase; GRK2, G protein-coupled receptor kinase 2; ERK, extracellular signal-regulated kinase; MMP-9, matrix metalloproteinase-9; JNK, c-jun kinase; AEDs, antiepileptic drugs; MCS, minimal clonic seizures; GTCS, generalized tonic-clonic seizures; GABAA-benzodiazepine receptor, γ-aminobutyric acid A-benzodiazepine receptor; CSE, hydro-ethanolic saffron extract; PSPs, postsynaptic potentials; PTSD, Post-traumatic stress disorder; CHR, corticotropin-releasing hormone; CBD, deep brain stimulation; NIHSS, National Institute of Health Stoke Scale; GABA, γ-aminobutyric acid
